# Epidemiological, Clinical and Virological Characteristics of Influenza B Virus from Patients at the Hospital Tertiary Care Units in Bangkok during 2011-2014

**DOI:** 10.1371/journal.pone.0158244

**Published:** 2016-07-07

**Authors:** Navin Horthongkham, Niracha Athipanyasilp, Archiraya Pattama, Bualan Kaewnapan, Suthatta Sornprasert, Surangrat Srisurapanont, Wannee Kantakamalakul, Palanee Amaranond, Ruengpung Sutthent

**Affiliations:** 1 Department of Microbiology, Faculty of Medicine, Siriraj Hospital, Mahidol University, Bangkok, Thailand; 2 Department of Pathology, Faculty of Medicine, Srinakharinwirot University, Bangkok, Thailand; 3 Department of Blood transfusion, Faculty of Allied Health Science, Chulalongkorn University, Bangkok, Thailand; Centers for Disease Control and Prevention, UNITED STATES

## Abstract

Influenza B virus, which causes acute respiratory infections, has increased in prevalence in recent years. Based on the nucleotide sequence of the hemagglutinin (HA) gene, influenza B virus can be divided into two lineages, Victoria and Yamagata, that co-circulate during the influenza season. However, analysis of the potential association between the clinical and virological characteristic and the lineage of influenza B viruses isolated in Thailand was lacking. To investigate influenza B virus genetically and determine its neuraminidase (NA) inhibitor susceptibility phenotype, a total of 6920 nasopharyngeal-wash samples were collected from patients with influenza-like illness between the years 2011 and 2014 and were screened for influenza B virus by real-time PCR. Of these samples, 3.1% (216/6920) were confirmed to contain influenza B viruses, and 110 of these influenza viruses were randomly selected for nucleotide sequence analysis of the HA and NA genes. Phylogenetic analysis of the HA sequences showed clustering into various clades: Yamagata clade 3 (11/110, 10%), Yamagata clade 2 (71/110, 64.5%), and Victoria clade 1 (28/110, 25.5%). The analysis of clinical characteristic demonstrated that the Victoria lineage was significantly associated with the duration of hospitalization, number of deceased cases, pneumonia, secondary bacterial infection and underlying disease. When combined with phylogenetic analysis of the NA sequences, four samples showed viruses with reassortant sequences between the Victoria and Yamagata lineages. Statistical analysis of the clinical outcomes and demographic data for the reassortant strains did not differ from those of the other strains in circulation. Oseltamivir-resistant influenza B viruses were not detected. Our findings indicated the co-circulation of the Victoria and Yamagata lineages over the past four cold seasons in Bangkok. We also demonstrated differences in the clinical symptoms between these lineages.

## Introduction

Influenza viruses comprise three members: influenza A, influenza B, and influenza C. Influenza A and B viruses are associated with annual worldwide epidemics. Influenza B virus cannot be classified into distinct subtypes; however, based on genetic analysis it can be divided into two lineages, denoted B/Victoria/2/87 and B/Yamagata/16/88 [1, 2]. Both lineages were first detected in 1988–1989 and co-circulated globally in the 1990s, with the Yamagata lineage being predominant [3–5]. During 2000–2002, the Victoria lineage became predominant worldwide [6]. Thus, the World Health Organization recommended strain B/Hong Kong/330/2001 (Victoria lineage) to be used as the vaccine strain during 2002–2003 [7, 8]. The level of cross protection offered by antibodies between two strains of influenza B virus was investigated but not detected [9].

In Thailand, from 2004 to 2010 influenza B virus strains circulated; B/Victoria and B/Yamagata strains co-circulated between 2004 and 2008 and B/Victoria strains predominated between 2009 and 2010 [10]. All of the strains that emerged matched the vaccine strain. In 2014, the Victoria and Yamagata lineages were still circulating in Thailand; however, some additional strains, B/Brisbane/60/2008 and B/Wisconsin/01/2010, which did not match the vaccine strains (in the Northern and Southern hemispheres), were also isolated [10]. This might present a problem for vaccine efficacy in the future.

The neuraminidase inhibitors oseltamivir and zanamivir have been used for the treatment of influenza B virus infection. In 2010, a reduction in the oseltamivir susceptibility of influenza B virus was reported in the USA [11]. This reduced susceptibility was confirmed by other studies of influenza B virus worldwide [12–14], and a new mutation was identified that was proposed to be responsible [12–14]. Since 2011, Thai guidelines advised physicians to prescribe antivirals for influenza as soon as possible in cases of influenza-like illness with pneumonia, in patients with severe symptoms, those with an increased risk of complications, and patients with non-severe symptoms that showed no improvement after 48 hours of other treatment [15]. Oseltamivir has been used widely for prophylaxis and treatment of influenza A pdm09 (H1N1) during outbreaks, especially in urban areas of Thailand but there are no reports of the actual number of cases treated [16]. As a result of the wide spread use of oseltamivir in Thailand, an increasing number of resistant strains has been demonstrated especially influenza A strain pdm09(H1N1) [16]. Oseltamivir resistant strains of influenza B virus were not detected during 2008–2010 [17], and study into oseltamivir resistant strains of influenza B virus in Thailand were lacking during 2011–2014.

The aims of the current study were to determine the genetic diversity of circulating influenza B viruses in Bangkok during 2011–2014. The association of clinical characteristics and influenza B virus lineages were investigated. Finally, mutation analysis of the neuraminidase (NA) gene and phenotypic assays for NA activity provided insight into oseltamivir drug resistance.

## Materials and Methods

### Sample collection and cell culture

A total of 6920 nasopharyngeal-wash samples were collected from patients with influenza-like illness at Siriraj hospital from 2011 to 2014. Influenza B virus identification was performed using a Proflu^+^ assay (Genprobe Bedford, MA, USA), following extraction performed according to the manufacturer’s instructions. Influenza B-positive samples were subjected to virus isolation using Mardin–Darby canine kidney (MDCK) cells. Cells were cultured and passaged once or twice in DMEM supplemented with 10% fetal bovine serum and the appropriate antibiotic. Then, a hemagglutination assay was performed to determine the concentration of hemagglutinin(HA) antigen for the NA inhibitor assay.

### Ethics statement

Our study was performed with nasopharyngeal washes which were stored as anonymous. The Institutional Review Board of the Faculty of Medicine Siriraj Hospital, Mahidol University approved the research protocol (COA: si148/2013). The IRB waived the need for consent due to all specimens were anonymous.

### Hemagglutinin (HA) and neuraminidase (NA) amplification

The NucliSens nucleic acid extraction kit (Biomeriux, Marcy l’Etoile, France) was used to extract influenza B virus RNAs from 200 μl of nasopharyngeal-wash. Extraction was performed according to the manufacturers’ instructions. Viral RNA was eluted with 80 μl of elution buffer. Then, cDNAs were generated using superscript III reverse transcriptase (Invitrogen, Carlsbad, CA, USA) and the Oligo-dT primer. The HA and NA genes were amplified using influenza B-specific primers.[S1 Table] Briefly, the HA gene was amplified by nested PCR using the HA42/HA1764 primers for the primary PCR and PrimeStar GXL polymerase (Takara, Ohtsu, Shiga, Japan). The first round amplification conditions were as follows: 35 cycles of 15 s at 98°C, 30 s at 50°C, and 2 min at 68°C, followed by 10 min at 68°C, with subsequent holding at 4°C. Nested PCR was performed using 5 μl of the first round product and the HA55/HA1742 primers. The nested PCR amplification conditions were as follows: 35 cycles of 15 s at 98°C, 30 s at 50°C, and 2 min at 68°C, followed by 10 min at 68°C, with subsequent holding at 4°C. Similarly, the NA gene was amplified by nested PCR using the NA22/NA1536 primers for the primary PCR. The first round amplification conditions were as described above. Nested PCR was performed using 5 μl of the first round product, the NA46/NA1473 primers, and the nested PCR amplification conditions described above. The sizes of the HA- and NA-amplified products were 1,688 and 1,428 base pairs, respectively. The PCR products were purified using the PureLink^®^ PCR purification kit (Invitrogen) and quantified using the Nanodrop technique.

### Sequence analysis

Sequencing was performed using the BigDye terminator v3.1 cycle sequencing kit and an ABI 3130 genetic analyzer (Applied Biosystems, Foster City, CA, USA). Nucleotide sequences were edited using DNAbaser v5.3.2 software and were aligned using Molecular Evolutionary Genetics Analysis software (MEGA, version 6.0; http://www.megasoftware.net/). A phylogenetic tree was constructed using the maximum likelihood method (1,000 replicates of bootstrap values) along with the MEGA version 6.0 programs. Influenza B virus vaccine strains were retrieved from the EpiFluTM DATABASE of the Global initiative on Sharing All Influenza Data (GISAID, http://www.gisaid.org) [18].

### GenBank accession numbers

The nucleotide sequences determined in this study were submitted to the GenBank database and assigned the following accession numbers: KF305428-KF305474, KF699250-KF699309 and KX058891- KX059012.

### Neuraminidase (NA) inhibition assay

The NA inhibition assay was carried out to determine the 50% inhibitory concentration (IC50) using the Neuraminidase Inhibitor Resistance Detection Kit (Applied Biosystems) according to the manufacturer’s recommendations. To determine the NA activity of the virus on oseltamivir, 25 μl of each oseltamivir carboxylate dilution was mixed with 25 μl of diluted virus and incubated at room temperature for 30 min. The reaction was terminated by the addition of 60 μl of accelerator solution. The luminescence was then read immediately using a luminometer (Perkin-Elmer). The IC50 of NA activity was determined using the GraphPad Prism 5.01 software (GraphPad Software, San Diego, CA, USA).

### Statistical analysis

Clinical characteristics between patients infected with influenza B viruses of the Victoria and Yamagata lineages were compared and analyzed using the Person chi-square test. Lineages were compared using the Mann–Whitney U test and the independent t-test. Fisher exact probability test was used to measure the association between two small populations (n ≤ 5). Statistical significance was set at *p* < 0.05.

## Results

### Prevalence of influenza B virus

**Fig 1:**
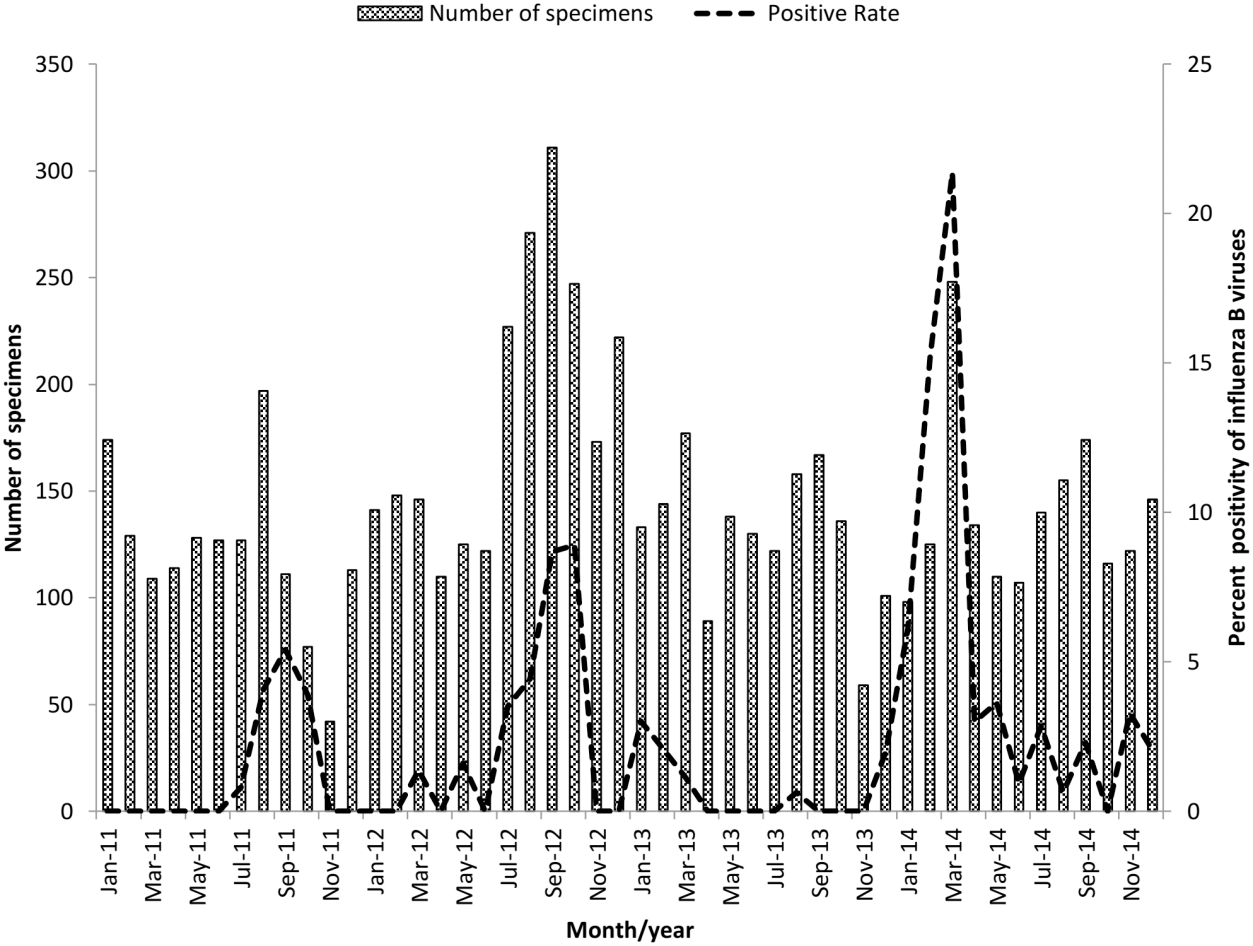
Monthly distribution of influenza B viruses specimens from patients in Thailand from January 2011 to December 2014. Total number of specimens are indicated by a bar and the positive rate of detection of influenza B virus is shown by a dotted line.

During 2011–2014, a total of 6920 nasopharyngeal-wash specimens were collected from patients with influenza-like illness and were tested for the presence of influenza virus. Of these specimens, 216 (3.1%) were confirmed to contain influenza B virus by real-time PCR. The rates of influenza B virus detection among our samples by year were 1% (18/1448), 3.7% (83/2243), 0.7% (11/1554), and 6.2% (103/1675) for the years 2011–2014, respectively. The 216 patients that tested positive for influenza B virus were aged from newborn to 93 years; mean, 23.54 years; and median, 9 years. Patients aged between 5–14 years were most frequently affected by influenza B virus (42.1%), followed by those aged newborn through to 4 years (25%). Of the influenza B-positive patients, 105 (48.6%) were male and 111 (51.4%) were female. The demographic data are presented in Table 1. The highest rates of influenza B virus detection were in September 2011, September 2012, January 2013 and March 2014 (Fig 1).

**Table 1:** Demographic data and the characteristics of influenza B virus-infected patients in Bangkok from 2011 to 2014.

		Determination of lineage (n = 110)		*P value
Characteristics		Victoria (n = 28)	Yamagata (n = 82)	
**Demographic feature**				
Mean age in years	23.54±28.3	25.75±5.76	16.74±2.52	0.068
Median in years	9	9.5	8	
**Number of patients aged between:**				
0–4	25% (54/216)	25% (7/28)	20.7% (17/82)	0.637
5–14	42.1% (91/216)	39.3% (11/28)	59.8% (49/82)	0.060
15.34	6% (13/216)	7.1% (2/28)	6.1% (5/82)	0.845
35–64	10.7% (23/216)	7.1% (2/28)	4.9% (4/82)	0.649
≥65	16.2% (35/216)	21.5% (6/28)	8.5% (7/82)	0.068
**Sex**				
Male	48.6% (105/216)	50% (14/28)	47.6% (39/82)	0.824
Female	51.4% (111/216)	50% (14/28)	52.4% (43/82)	0.824
**Clinical features**				
Body temperature	38.7°C	39.0°C	38.6°C	0.36
Duration of hospitalization	6 days	12.25 days	3.68 days	<0.05
Deceased cases	10% (11/110)	21.4% (6/28)	6.1% (5/82)	<0.05
Oseltamivir treatement	77.3% (85/110)	57.1% (16/28)	84.1% (69/82)	<0.05
Vaccination	4.5% (5/110)	0% (0/28)	6.1% (5/82)	0.181
**Symptoms**				
Cough	68.2% (75/110)	39.3% (11/28)	78% (64/82)	<0.05
Runny nose	42.7% (47/110)	25% (7/28)	48.8% (40/82)	<0.05
Fatigue	23.6% (26/110)	17.9% (5/28)	25.6% (21/82)	0.404
Wheezing	1.8% (2/110)	3.6% (1/28)	1.2% (1/82)	0.446
**Lower respiratory infection**				
Pneumonia	10.9% (12/110)	25% (7/28)	6.1% (5/82)	<0.050
Bronchitis	2.7% (3/110)	3.6% (1/28)	2.4% (2/82)	1
Respiratory failure	3.6% (4/110)	7.1% (2/28)	2.4% (2/82)	0.268
**Bacterial infection**	11.8% (13/110)	21.4% (6/28)	8.5% (7/82)	<0.05
**Underlying disease**	48.2% (53/110)	71.4% (20/28)	40.2% (33/82)	<0.05

*p values were calculated using the Mann–Whitney U test and the independent t-test.

### Phylogenetic analysis of the HA gene

**Fig 2:**
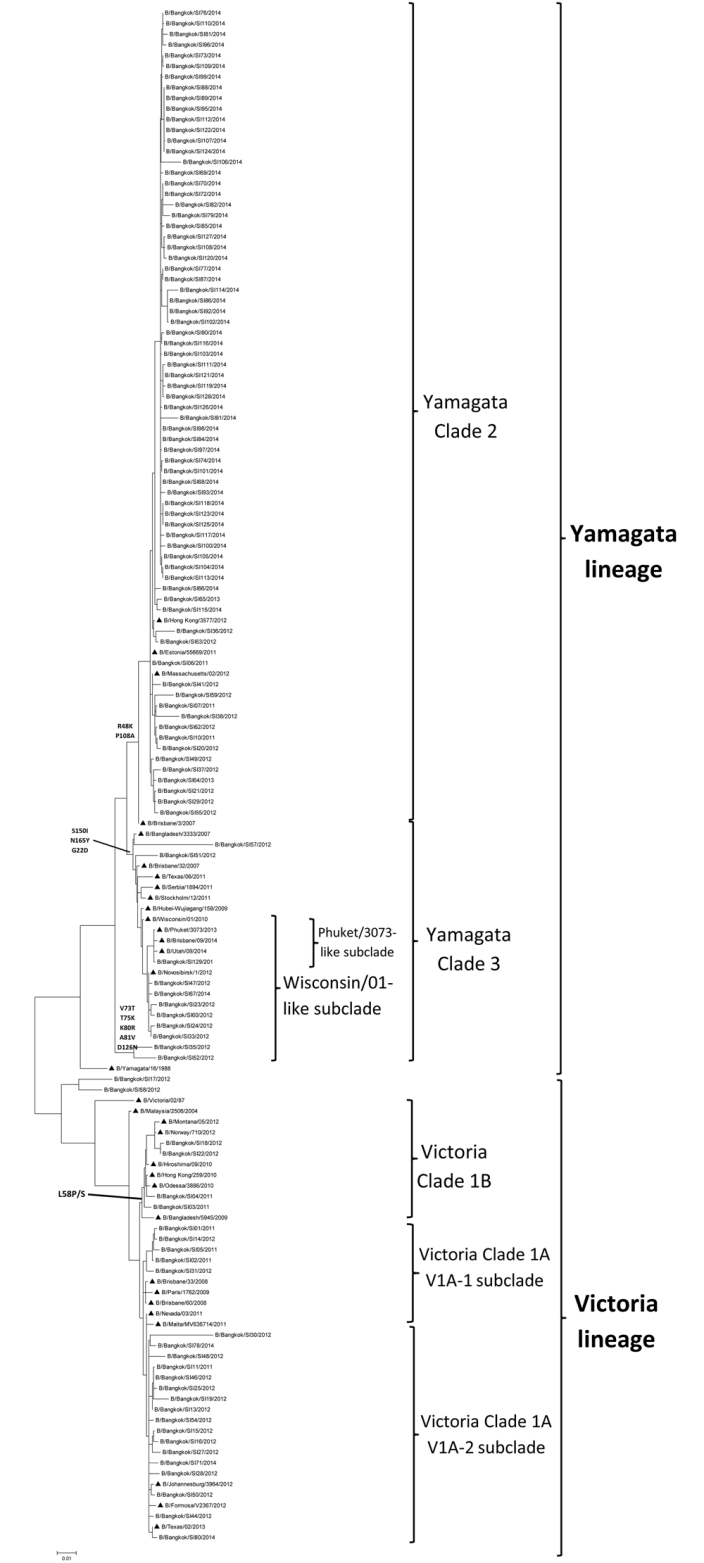
Phylogenetic tree of the HA gene sequences of influenza B viruses. ▲ represents the vaccine strain.

**Fig 3:**
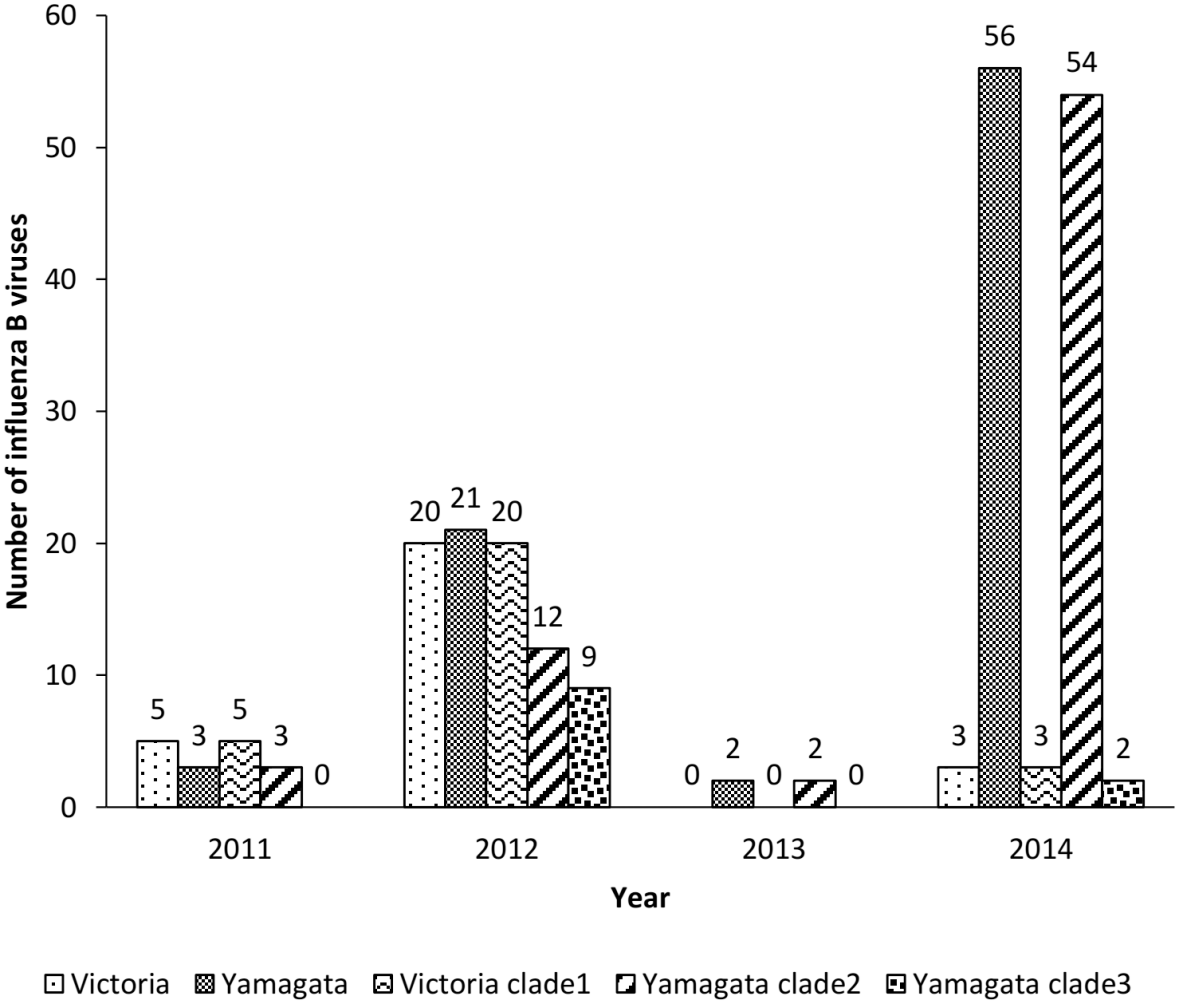
Yearly distribution of influenza B viruses, isolated in Thailand from 2011 to 2014, by lineage.

Influenza B viruses were classified into subgroups (Yamagata and Victoria lineages) based on their HA gene sequence. To determine the subgroups of the viruses, a total of 110 partial HA and NA sequences were randomly selected from 216 positive influenza B virus clinical samples. Phylogenetic analysis of the HA sequences showed that 25.5% (28/110) of influenza B viruses from 2011 to 2014 clustered in the Victoria-like lineage, while 74.5% (82/110) of influenza B viruses clustered in the Yamagata lineage (Fig 2, Table 1). The distribution of viruses belonging to the Victoria lineage by year from 2011 to 2014 was 62.5% (5/8), 48.8% (20/41), 0% (0/2), and 5.1% (3/59), respectively (Fig 3). Phylogenetic analysis can further classify the Victoria lineage into clade 1A (subclades V1A-1 and V1A-2) and clade 1B. Victoria clade 1A was found to be predominant among the viruses circulating in Thailand from 2011 to 2014 and this related to influenza B virus vaccine strain B/Brisbane/60/2008. Analysis of Victoria clade 1B identified amino acid changes at position L58P/S/D. The prevalence of viruses of the Yamagata lineage increased year by year during the period 2011–2014, replacing Victoria lineage viruses by 37.5% (3/8), 51.2% (21/41), 100% (2/2), and 94.9% (56/59), respectively (Fig 3). Phylogenetic analysis of the HA amino acid sequence classified viruses belonging to the Yamagata lineage into two clades, clade 2 and 3. Viral strains belonging to Yamagata clade 3 shared amino acids substitutions at S150I, N165Y, and G22D, whereas Yamagata clade 2 shared amino acids substitutions at T181A and D196N. From 2011 to 2014, Yamagata clade 2 increased in prevalence among circulating viruses, becoming predominant in 2014. Yamagata clade 2 was related to the influenza B virus vaccine strain B/Massachusetts/02/2012, whereas clade 3 was related to strain B/Wisconsin/01/2010.

### Phylogenetic analysis of the NA gene

**Fig 4:**
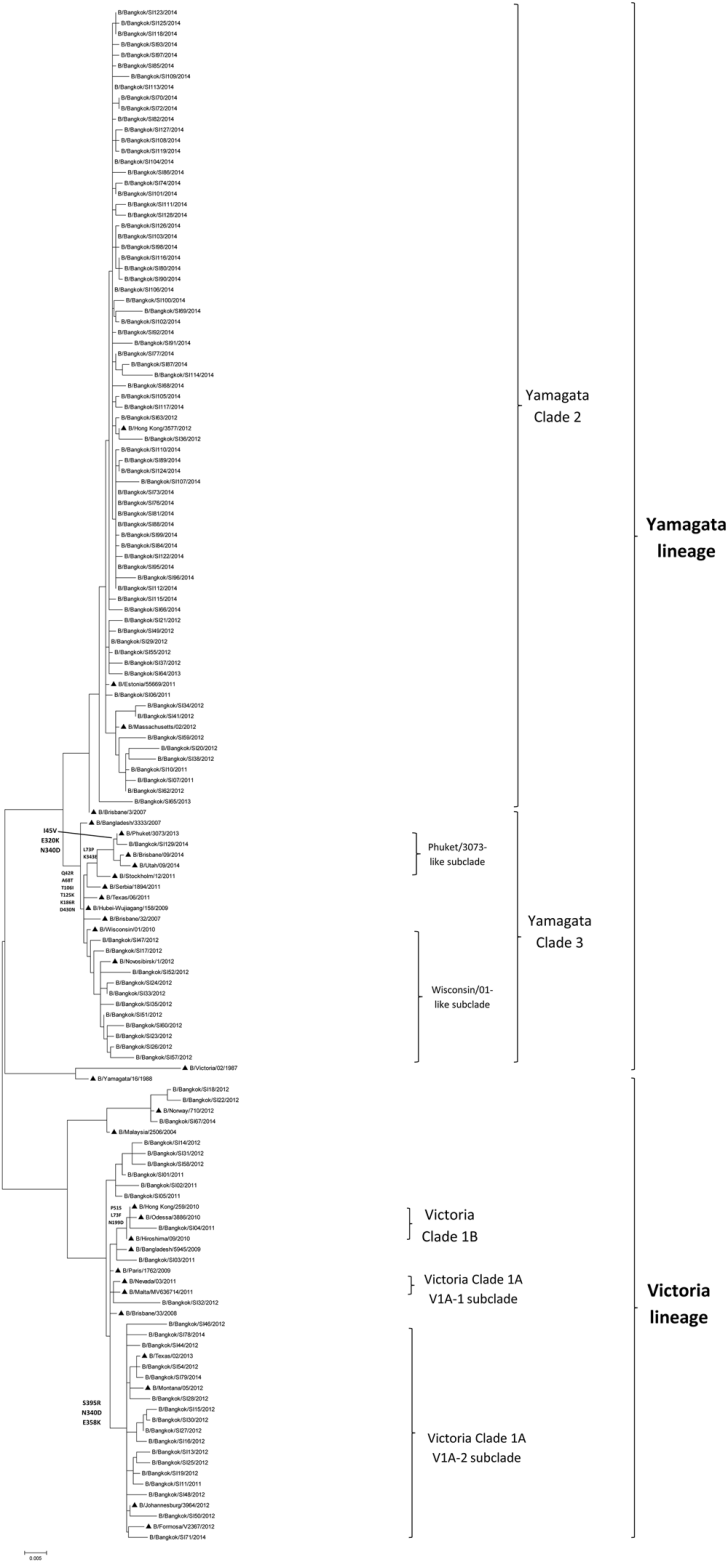
Phylogenetic tree of the NA gene sequences of influenza B viruses. ▲ represents the vaccine strains.

Phylogenetic analysis of the NA gene from influenza B virus-positive specimens isolated during 2011–2014 indicated that 28 of the viral isolates clustered in the Victoria-like lineage and 82 clustered in the Yamagata lineage (Fig 4). During 2011–2014, viruses of the Victoria-like lineage were circulating at a prevalence of 62.5% (5/8), 48.8%(20/41), 0%(0/2), and 5.1%(3/59), respectively. The Victoria lineage can be classified into clades 1A and 1B according to the NA gene, and most Victoria clade 1A viruses were isolated during 2012. Clade 1A can be further classified into subclades V1A-1 and V1A-2. Subclade V1A-2 shared amino acids at positions S39R, N340D, and E385K, whereas clade 1B shared amino acids at positions P51S, L73F, and N199D. During 2011–2014, viruses belonging to the Yamagata lineage were circulating at a prevalence of 37.5% (3/8), 51.2% (21/41), 100% (2/2), and 94.9% (56/59), respectively. Similarly, the Yamagata lineage can be subdivided into clades 2 and 3. Clade 3 shared amino acids at positions Q42R, A68T, T106I, T125K, K186R, and D340N. Most viral specimens isolated in 2013 and 2014 belonged to clade 2 of the Yamagata lineage, which was related to influenza B virus vaccine strain B/Massachusetts/02/2012. Clade 2 can be classified into two subclades, Phuket/3073-like and Wisconsin/01-like. Subclade Phuket/3073-like shared amino acids at positions L73P and K343E.

### Reassortant detection

From HA and NA analysis, we could detect reassortant strains between Victoria lineage and Yamagata lineage. Four viral isolates showed reassortment between the HA and NA genes (B/Bangkok/SI17/2012, B/Bangkok/SI67/2014, B/Bangkok/SI79/2014, and B/Bangkok/SI80/2014). For two of these isolates, the HA gene belonged to the Victoria lineage and the NA gene belonged to the Yamagata lineage (B/Bangkok/SI17/2012 and B/Bangkok/SI80/2014). For the other two isolates, the HA gene belonged to the Yamagata lineage and the NA gene belonged to the Victoria lineage (B/Bangkok/SI67/2014 and B/Bangkok/SI79/2014). One of these viruses was isolated in 2012, whereas the others were isolated in 2014.

### Demographics and clinical characteristics of the patients infected with different lineages of influenza B virus

To compare the characteristics associated with influenza B virus lineages, demographic data and clinical information were analyzed, as shown in Table 1. The average age and sex of patients infected with influenza B virus did not differ significantly between the Yamagata and Victoria lineages (*p*>0.05). Similarly, body temperature and vaccination status did not differ significantly between the two lineages (*p*>0.05). However, the number of deceased cases did differ significantly between the two lineages (*p*<0.05). All 11 (10%) of the deceased patients had a history of underlying disease and most of them were older (70–93 years), with the exception of one 16-year-old. By contrast, the duration of hospitalization among patients infected with viruses of the Victoria lineage was greater than for those infected with Yamagata lineage viruses (*p*<0.05). Furthermore, the number of patients who received oseltamivir treatment was higher with viruses of the Yamagata lineage than with viruses of the Victoria lineage (*p*<0.05). Secondary bacterial infection was greater with viruses of the Victoria lineage than with viruses of the Yamagata lineage (*p*<0.05). The most common secondary bacterial infection after influenza B virus infection was *Pseudomonas aeuroginosa* followed by *Staphylococcus aureus*, *Acinetobacter baumannii*, *Escherichia coli* (ESBL), and *Klebsiella pneumonia*e. In general, the symptoms associated with the two lineages did not differ significantly, except that a cough and runny nose were more frequently associated with the Yamagata lineage (*p*<0.05). For 53 of the influenza B-infected patients, underlying diseases were reported that included acute lymphoblastic leukemia, allergic rhinitis, anemia, asthma, beta-thalassemia/HbE, disseminated intravascular coagulation, diabetes type 2, and heart disease. The existence of underlying diseases in patients was more frequently associated with infection with viruses of the Victoria lineage (20/28, 71.4%) than the Yamagata lineage (33/82, 40.2%) (*p*<0.05). Similarly, pneumonia was more common among patients infected with viruses of the Victoria lineage (7/28, 25%) than the Yamagata lineage (5/82, 6.1%). The analysis of reassortant virus-infected patients showed no difference in clinical symptoms or history of underlying disease. None of the reassortant virus-infected patients died and only one was admitted to hospital (for 5 days).

#### Fatal cases

In terms of illness severity, 11/110 (10%) patients died during hospitalization (Table 2). The mean and median ages of the deceased cases were 73.5 and 77 years, respectively. The median number of days between admission and death was 12 days. Based on the medical records, among these patients, 8 of 11 had secondary bacterial infections, and 3 of 11 suffered cardiac arrest and acute respiratory failure. All of the deceased cases had been treated with oseltamivir for at least 2 days during their hospitalization, and only one patient had a documented history of influenza vaccination. Among the deceased cases there were no significant difference in the lineage of causative virus; six patients were infected with Victoria lineage viruses and five patients were infected with Yamagata lineage viruses. All deceased patients shared hypertension as an underlying disease. Most of the deceased patients were infected with bacteria after influenza B virus infection, with the exception of three 3 cases. Only three cases were diagnosed with influenza pneumonia.

**Table 2:** Demographic and clinical characteristics of fatal patients during 2011–2014.

Influenza B virus	Influenza B virus lineage	Age	Duration of hospitalization(days)	Oseltamivir treatment received (days)	Vaccination	Underlying disease	Cause of death	Bacterial infection	Complications
B/Bangkok/SI03/2011	Victoria 1B	84	18	5	unknown	Hypertension	pneumonia	*Acinenobacter baumannii*,	Septic shock
B/Bangkok/SI05/2011	Victoria 1A-2	82	39	5	unknown	Hypertension	Salmonella enteritis	*Acinenobacter baumannii*, *Staphylococcus aureus*	Pneumonia with bacteria
B/Bangkok/SI13/2012	Victoria 1A-1	81	12	5	unknown	Hypertension	Influenza with pneumonia	No	Septicemia, acute respiratory failure
B/Bangkok/SI23/2012	Yamagata 3	93	9	5	unknown	Hypertension, chronic heart disease	Influenza with pneumonia	*Acinenobacter baumannii*	Pneumonia with bacteria
B/Bangkok/SI25/2012	Victoria 1A-1	74	25	5	unknown	Hypertension, heart disease	Influenza with pneumonia	*Acinenobacter baumannii*	Pneumonia with bacteria
B/Bangkok/SI29/2012	Yamagata 2	77	2	2	unknown	Hypertension, DM, dementia	Acute bronchitis	No	Cardiac arrest
B/Bangkok/SI35/2012	Yamagata 3	76	6	5	Yes	Hypertension, coronary artery disease	Influenza with pneumonia	*Staphylococcus aureus*	Acute respiratory failure
B/Bangkok/SI36/2012	Yamagata 2	74	6	5	unknown	Hypertension	Sepsis (UTI)	*Pseudomonas aeruginosa*	Acute respiratory failure
B/Bangkok/SI50/2012	Victoria 1A-1	70	3	3	unknown	Hypertension, DM, heart disease	Myocarditis infarction	No	Ventricular fibrillation
B/Bangkok/SI58/2012	Victoria 1B	16	102	5	unknown	Hypertension	Septic shock	*Pseudomonas aeruginosa*	Candidiasis sepsis
B/Bangkok/SI62/2012	Yamagata 2	81	44	5	No	Hypertension, ischaemic stroke	Septic shock	*Pseudomonas aeruginosa*	Pneumonia with bacteria

### Amino acid analysis of the HA and NA sequences of influenza B viruses

Wang *et al*. reported four major antigenic epitopes on the membrane distal domain of HA1(a subunit of HA): the 120 loop (position 116–137 of HA1), the 150 loop (position 141–150), the 160 loop (position 162–167), and the 190 helix (position 194–202)[19]. In our study, mutation analysis was performed in these regions by comparing the sequences to those of influenza B virus strain.

B/Hong Kong/8/1973. In the 120 loop, Victoria lineage clade 1 revealed amino acid substitutions at positions Q48E, N56K, T75K, N116H, R122H, T125I, T129N, and TKG179–181TEG, while Yamagata clade 2 showed amino acid substitutions at positions Q48K, N56D, K71M, R122Q, T125I, T129K, and TKG179–181AEG, as shown in Table 3. In the 150 loop, no amino acid substitutions were observed in Victoria lineage clade 1, while Yamagata clades 2 and 3 showed amino acid substitutions at positions NGN148–150SKS and NGN148–150SKI, respectively. In the 160 loop, Victoria clade 1 showed a DKN insertion at position 162–163, while Yamagata clades 2 and 3 showed amino acid insertions of NN and NY, respectively. In the 190 loop, Victoria clade 1 showed amino acid substitutions at positions V199A, A232T, and E235G, while Yamagata clades 2 and 3 both showed amino acid substitutions at positions E195K, V199K, K206N, N230D, and E235G, except for position 232 where clade 2 showed A232R and clade 3 showed A232T. The site for receptor binding was also altered, the substitution I136K was evident in the Victoria lineage, and I136R was observed in the Yamagata clade 2 and 3 sequences. A potential glycosylation site at amino acid position D194N (based on the sequence of strain B/Hong Kong/08/1973) was evident in all viral sequences isolated from 2011 to 2014 and the influenza B virus vaccine strains (B/Brisbane/60/2008, B/Wisconsin/01/2010, and B/Phuket/3073/2013), with the exception of strain B/Massachusetts/02/2012.

**Table 3:** Amino acid substitutions in the antigenic sites of isolated influenza B viruses.

Amino acid position*	Victoria clade 1 (%)	Yamagata clade 2 (%)	Yamagata clade 3 (%)
**120 loop**			
48	E(96.3%), Q(3.7%)	K(98.6%), E(1.4%)	R(100%)
56	K(96.3%), D(3.7%)	D(97.2%), N(1.4%), Y(1.4%)	D(90.9%), N(9.1%)
71	K(100%)	M(98.6%), K(1.4%)	M(100%)
75	K(100%)	T(91.5%), N(8.5%)	T(82.8%), K(18.2%)
116	H(100%)	N(98.6%), H(1.4%)	K(82.8%), N(18.2%)
122	H(77.8%), Q(18.5%), Y(3.7%)	Q(98.6%), R(1.4%)	Q(90.9%), H(9.1%)
125	I(100%)	I(97.2%), M(1.4%), D(1.4%)	I(100%)
129	N(92.6%), T(3.7%), K(3.7%)	K(94.4%), N(1.4%), S(1.4%), Q(2.8%)	K(82.8%), N(18.2%)
179–181	TEG(100%)	AEG(95.8%), AEE(2.8%), TEG(1.4%)	TEG(82.8%), TKE (9.1%), TEE(9.1%)
**150 loop**			
148–150	NGN(96.3%), NKN(3.7%)	SKS(98.6%), SRS(1.4%)	SKI (82.8%), SKS(9.1%), SRN(9.1%)
**160 loop**			
Insertion 162–163	NDKN(96.3%), NNKN(3.7%)	DNN(100%)	DNY(90.9%), DNN(9.1%)
**190 helix**			
195	E(100%)	K(100%)	K(100%)
199	A(96.3%), E(3.7%)	K(100%)	K(100%)
206	K(96.3%), N(3.7%)	N(100%)	N(100%)
230	N(100%)	D(100%)	D(100%)
232	T(100%)	R(74.6%), T(25.4%)	T(100%)
235	G(100%)	G(100%)	G(100%)
**Receptor binding site**			
136	K(96.3%), R(3.7%)	R(100%)	R(100%)

*Influenza B virus strain B/Hong Kong/8/1973

The NA protein is composed of eight catalytic residues (R116, D149, R150, R223, E275, R292, R374, and Y409) that interact with sialic acid and 11 framework residues (E117, R154, W177, S178, D197, I221, E226, H273, E276, N293, and E428) that support the enzymatic binding pocket. The catalytic residues and framework residues were conserved among all of the viruses isolated during 2011–2014. Furthermore, the residues responsible for resistance to NA inhibitor were not present in any of the isolates (position E117V/A, D197N/E/Y, I221T, H273Y, R292K, and R374K). Comparing amino acid residues in the two glycoproteins: HA and NA, we found no amino acid differences between the fatal and recovered cases.

### Susceptibility of influenza B virus to oseltamivir as analyzed by the 50% inhibitory concentration (IC50)

**Fig 5:**
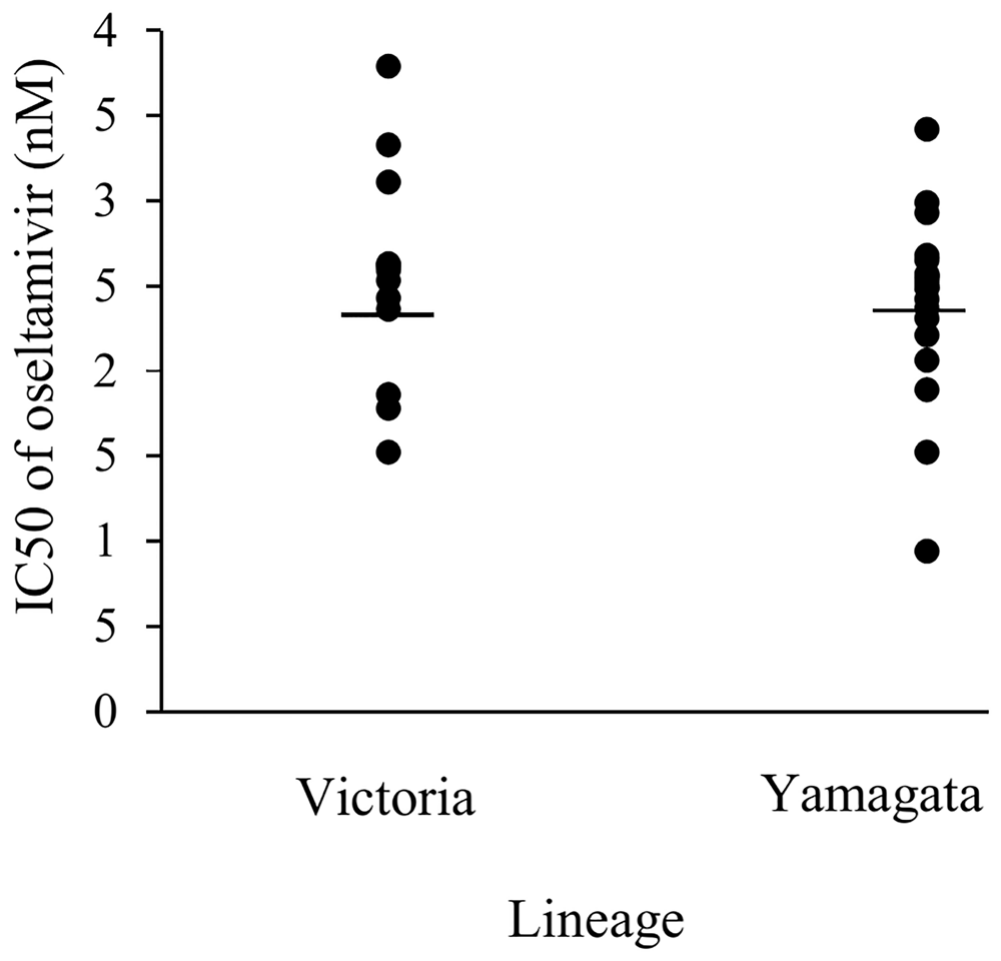
The 50% inhibitory concentration (IC50) of oseltamivir against influenza B virus isolates.

A total of 27 samples were available for influenza B virus isolation and were used for oseltamivir susceptibility testing using NA Star. A chemiluminescent substrate-based assay was used to determine the IC50 of oseltamivir against the influenza B virus isolates and all isolates showed susceptibility to oseltamivir within the range of 2.44±0.57 nM (compared with susceptible influenza B virus reference strain B/Lee/40 with an IC50 = 2.70 nM), as shown in Fig 5. The Victoria lineage (2.41±0.69 nM) exhibited similar IC50 values compared with the Yamagata lineage (2.45±0.54 nM).

## Discussion

Global circulation of influenza viruses including influenza A virus (pdm09 and H3N2) and influenza B virus was detected every year depending on the climate. In Thailand, the prevalence of influenza virus infections tends to increase during rainy season, especially during June–September. In general, seasonal influenza viruses are the most prevalent annually; however, in 2009, an outbreak of influenza A (H1N1) pdm09 virus occurred. Although influenza B virus is a minor circulating strain, an increase in the prevalence and severity of cases was reported during 2009–2012 [20–22]. In this study, the prevalence of influenza B virus during 2011–2014 was found to be 3.1%, which was concordance to previously report for 2010–2014 (3.27%) [23]. Influenza B virus infection was most prevalent among patients aged between 5 and 14 years (42.1%), in agreement with previous studies [23, 24]. Influenza B virus infections peaked in September 2011, September 2012, January 2013 and March 2014. Fluctuations in the prevalence of influenza A and B viruses were observed during 2011–2014, and when influenza A (H3) virus increased this was accompanied by a decrease in influenza A (H1N1) pdm09 virus and influenza B virus. In 2013, the lowest incidence of influenza B virus infection (0.7%) was detected, along with the highest incidence of influenza A (H3) virus infection (5.8%).

Phylogenetic analysis indicated that the HA sequences of influenza B virus determined in this study could be divided into two distinct lineages, the Victoria and Yamagata lineages. Although co-circulation of the Victoria and Yamagata lineages was found during 2011–2014, a switch in prevalence from the Victoria to the Yamagata lineage occurred in 2013 and 2014. This lineage shift had previously been reported in Malaysia and Thailand [23, 25]. The Victoria lineage can be subdivided into Victoria 1A and 1B. The Victoria 1A lineage was commonly detected in Thailand and Malaysia [23, 25]. The Yamagata lineage can be subdivided into clades 2 and 3. Yamagata clade 2 was found to gradually increase in prevalence from 2011 to 2013, then increase more significantly in 2014, confirming the findings of a previous study [23]. By contrast, in Malaysia, a shift from Yamagata 2 to Yamagata 3 during the 2012–2014 season was reported [25]. There were a few reports investigating the association between clinical characteristic and influenza B virus lineage, but no such studies were performed in Thailand [26]. In our study, a cough and runny nose were found to be more frequently associated with infection of viruses of the Yamagata lineage than the Victoria lineage, which was different from the result of Malaysian study [25]. In contrast, they demonstrated that nasal congestion and sore throat were associated to Victoria-lineage infection [25]. Furthermore, our study showed that both the Victoria and Yamagata lineages were more likely to infect patients aged between 5 and 14 years, which had also been observed in a previous study [24]. However, our finding also revealed differences in the Victoria lineage with regard to the duration of hospitalization, pneumonia, bacterial infection and underlying disease, which were not in accord with a previous study [27]. Of note, the 11 deceased cases in our study were predominantly infected with Yamagata lineage, clade 2 and Victoria lineage clade 1A-1. Our study partially confirmed a study from Taiwan that detected Yamagata lineage clade 2 viruses in deceased cases but not Victoria lineage viruses [28]. However, the Victoria lineage caused many more detrimental effects than the Yamagata lineage. Moreover, a higher number of patients infected with Victoria lineage viruses had underlying diseases, compared with those infected with Yamagata lineage viruses. The severe outcomes observed in those infected with Victoria lineage viruses could be due to the higher proportional of patients with pre-existing underlying diseases for the Victoria lineage.

Four major antigenic epitopes of the influenza B virus have been reported to be associated with HA at loops 120, 150, 160, and 190, and their surrounding regions [29–33]. Analysis of these four antigenic epitopes among our viral isolates revealed mutations in all epitopes, especially in the 120 loop. Many of these mutations had previously been reported, with the exception of mutations identified in the Yamagata clade 3 sequence that included N116K (120 loop), TEG179–181 (120 loop), and SKI148–150 (150 loop) [23, 34]. Moreover, we found an insertion within the 160 loop at position 162 NDKN or NNKN in the Victoria lineage that had not previously been reported. Insertions or deletions within the 160 loop region might enable influenza B virus to survive longer without undergoing an antigenic shift [35].

Phylogenetic analysis of the NA sequences from our viral isolates indicated the existence of two lineages, as was seen for the HA sequences. Only four viral isolates showed reassortant sequences, two displayed a HA gene of the Victoria lineage and an NA gene of the Yamagata lineage, while the other two displayed an NA gene of the Victoria lineage and a HA gene of the Yamagata lineage. Reassortment between different lineages has been reported in other studies in other countries [36–38]. However, regarding clinical outcomes, no significant differences were detected between reassortant viruses of the Victoria and Yamagata lineages. Moreover, our study has shown that amino acid of HA and NA was not different in fatal and recovered patients. This finding suggests that HA and NA gene may not involve in severe outcome.

Since 2011, the widespread use of oseltamivir for treatment or prophylaxis was advised in Thailand. Our study demonstrated that 77.3% of oseltamivir was used for the treatment of influenza B virus in hospitalized patients and outpatients. We investigated an influenza B oseltamivir-resistant strain by performing phenotypic and genetic analyses. Mutations associated with oseltamivir drug resistance were not detected in the NA gene of our viral isolates, which correlated with the results of our phenotypic assay.

The vaccine composition in 2013–2014 was changed from the Victoria lineage to the Yamagata lineage (B/Massachusetts/2/2012-like virus) due to the increase in prevalence of Yamagata lineage of influenza B virus in 2011 and 2012. Despite this, patients with Yamagata lineage (clade 2) influenza B virus infection were still increase in 2014. Our study has shown that 5/82 known vaccinated patients did have influenza B virus, Yamagata lineage infection. On the other hand, the other patients with unknown history vaccination (77/82) had this virus infection. This may indicate that these patients did not have flu vaccination, i.e., the coverage of influenza vaccine was low. In addition, the coverage may be lower than previous study in 2013 (30%) [39]. Moreover, there was no report of vaccine effectiveness in Thailand during 2013–2014.

In summary, this study details the circulation of influenza B viruses during 2011–2014 in Bangkok and reveals reassortant strains of the Victoria and Yamagata lineages. No significant association was found between clinical characteristics and influenza B virus lineages except cough and runny nose. However, severe outcome were found to be associated with pre-existing underlying diseases. We confirmed that influenza B viruses isolated during 2011–2014 retained susceptibility to neuraminidase inhibitor.

## Supporting Information

S1 Table Primer sets used for HA and NA amplification of influenza B strains. (TIF)
