# Supplementary figures and images for: Epidemiological, Clinical and Virological Characteristics of Influenza B Virus from Patients at the Hospital Tertiary Care Units in Bangkok during 2011-2014
Source: PLoS One. 2016 Jul 7;11(7):e0158244. doi: 10.1371/journal.pone.0158244 (PMC4936745; doi:10.1371/journal.pone.0158244)

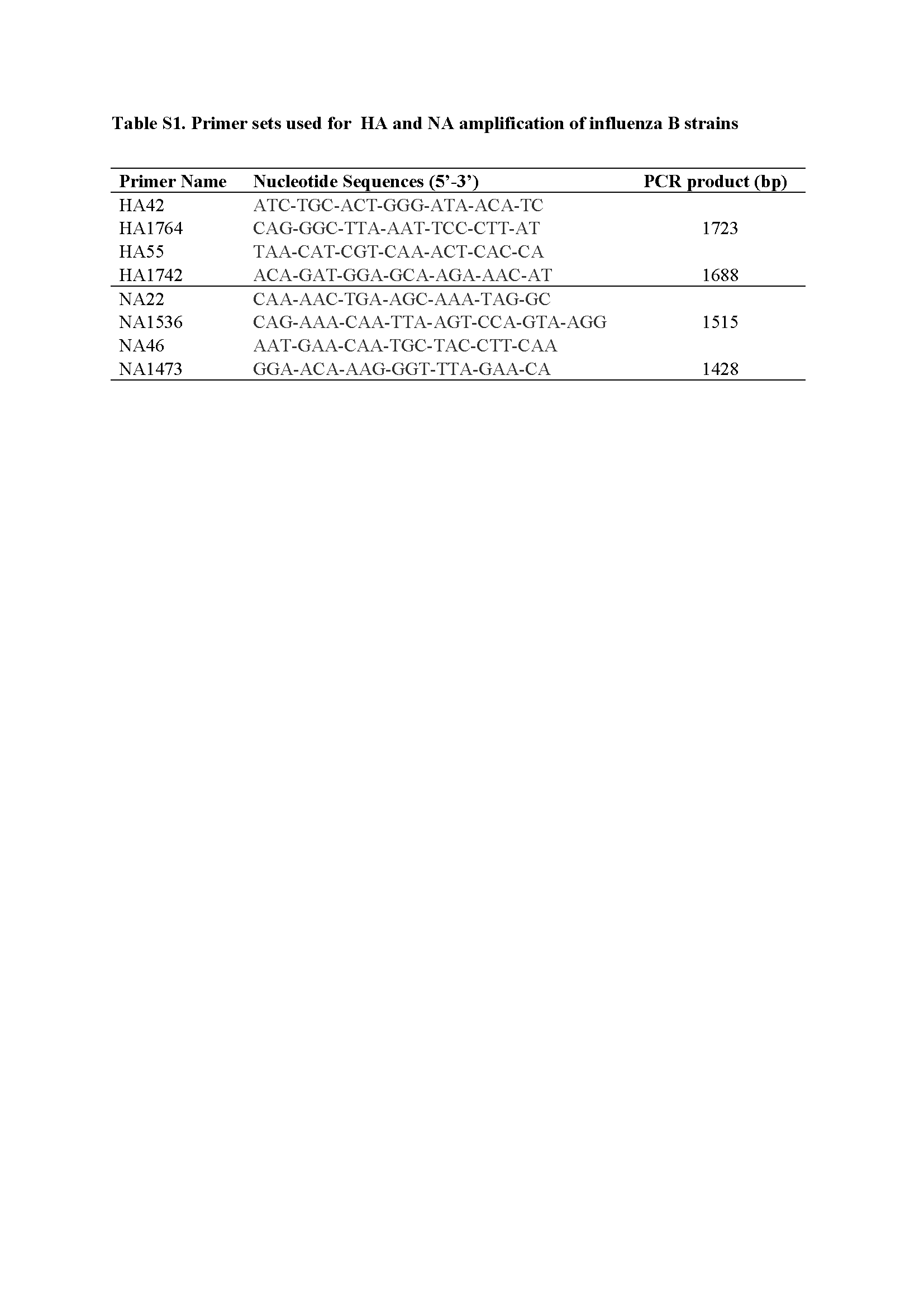

Supplement: S1 Table — (TIF) [file pone.0158244.s001.tif]
